# Reciprocal regulation of p63 by C/EBP delta in human keratinocytes

**DOI:** 10.1186/1471-2199-8-85

**Published:** 2007-09-28

**Authors:** Serena Borrelli, Barbara Testoni, Maurizio Callari, Daniela Alotto, Carlotta Castagnoli, Rose-Anne Romano, Satrajit Sinha, Alessandra M Viganò, Roberto Mantovani

**Affiliations:** 1 Dipartimento di Scienze Biomolecolari e Biotecnologie. U. di Milano. Via Celoria 26, 20133 Milano, Italy; 2 Dipartimento di Chirurgia Plastica-Banca della Cute, Ospedale CTO, Torino, Italy; 3 Department of Biochemistry, SUNY, Buffalo, USA

## Abstract

**Background:**

Genetic experiments have clarified that p63 is a key transcription factor governing the establishment and maintenance of multilayered epithelia. Key to our understanding of p63 strategy is the identification of target genes. We perfomed an RNAi screening in keratinocytes for p63, followed by profiling analysis.

**Results:**

C/EBPδ, member of a family with known roles in differentiation pathways, emerged as a gene repressed by p63. We validated C/EBPδ as a primary target of ΔNp63α by RT-PCR and ChIP location analysis in HaCaT and primary cells. C/EBPδ is differentially expressed in stratification of human skin and it is up-regulated upon differentiation of HaCaT and primary keratinocytes. It is bound to and activates the ΔNp63 promoter. Overexpression of C/EBPδ leads to alteration in the normal profile of p63 isoforms, with the emergence of ΔNp63β and γ, and of the TA isoforms, with different kinetics. In addition, there are changes in the expression of most p63 targets. Inactivation of C/EBPδ leads to gene expression modifications, in part due to the concomitant repression of ΔNp63α. Finally, C/EBPδ is found on the p63 targets *in vivo *by ChIP analysis, indicating that coregulation is direct.

**Conclusion:**

Our data highlight a coherent cross-talk between these two transcription factors in keratinocytes and a large sharing of common transcriptional targets.

## Background

p63 is a transcription factor -TF-homologous to the tumour suppressor p53 and to p73 [1]. This class of proteins activate and repress genes as a result of binding to promoters and enhancer regions. Six isoforms can be found as a result of different transcription initiation sites and alternative splicing. p63 proteins contain -TA- or lack -ΔN- a transcriptional activation domain at the N-terminal, and the sterile alpha motif -SAM- domain, presumably a protein-protein interaction module. The resulting proteins have dissimilar transcriptional properties and, as a result, different biological behaviour. The paramount importance of p63 in development has been broadly illustrated by genetic experiments in different organisms. Mice lacking p63 die soon after birth with severe defects in limb, craniofacial and skin development [2,3]. The major isoform present in keratinocytes and other epithelia -ΔNp63α- is essential for ectodermal development in zebrafish [4]. In humans, several syndromes showing abnormalities in limbs, skin and annexes are caused by mutations in the p63 gene [5]. In general, therefore, p63 is essential for the biology of multilayered epithelia.

Several TFs have an established role in keratinocytes programs [6], but few of them have so far been linked to p63 regulation. This topic is quite relevant since renewal of stem cells and ongoing active terminal differentiation most likely requires the progressive fine tuning of transctriptional programs, unlikely to be masterminded by a single TF.

C/EBPs are a family of six B-Zip TFs that activate and repress transcription under different differentiation and growth arrest conditions [7]. C/EBPα and C/EBPβ are required for differentiation of adipocytes [8-10]. Inactivation of C/EBPε in KO mice leads to lack of natural killer cells [11]. C/EBPδ KO has a mammary phenotype, with an alteration in the involution of the mammary glands upon lactation [12,13]. Adipocyte differentiation is also impaired in cells that lack C/EBPδ when cultured *in vitro*, similarly to C/EBPβ [14].

By inactivating p63 in HaCat and primary cells, it was possible to investigate the roles of p63 in keratinocytes, notably the importance for differentiation and cell adhesion programmes [15,16]. At the same time, however, p63 plays a major role in maintaining the proliferative potential of stem cells of the multilayered epithelia [1]. Through the use of RNAi inactivation coupled to gene expression profiling, as well as ChIP on chip experiments, several labs have recently identified hundreds of p63 targets [15-20]. Specifically, C/EBPδ emerged in the RNAi profiling of human HaCaT cells and primary keratinocytes as a target of p63 [18,19]. This prompted us to validate it and characterize the p63-C/EBPδ connections by RT-PCR, ChIPs and immunofluorescence in human keratinocytes.

## Results

### Validation of C/EBPδ as a target of p63

**Figure 1:**
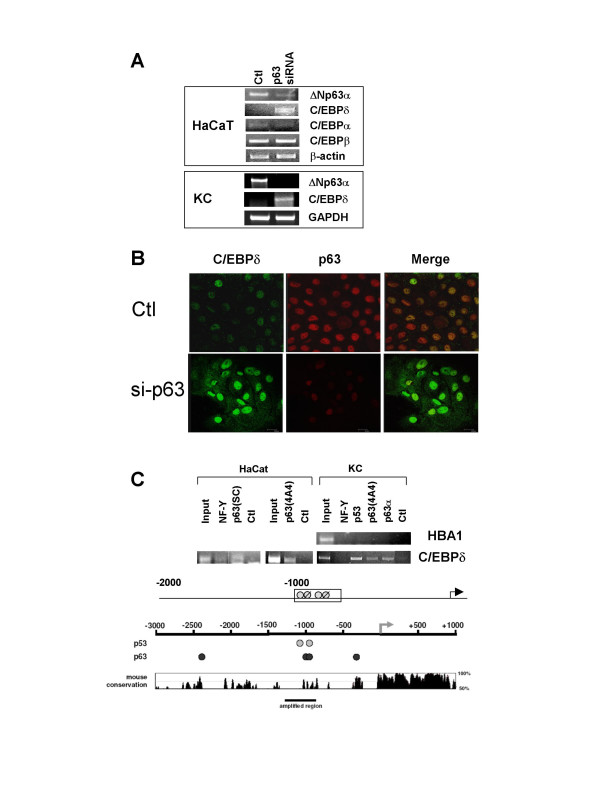
**Validation of C/EBPδ as a target of p63**. **A**. Evaluation of C/EBPα, C/EBPβ and C/EBPδ, by semi-quantitative RT-PCR analysis in control and cells treated with siRNA of p63. β-actin was used as an internal control. In the lower Panel, primary human keratinocytes (KCs) were treated similarly and RT-PCR performed for ΔNp63α and C/EBPδ; the invariant GAPDH was included as internal control. **B**. Confocal microscopy immunofluorescence analysis of primary KCs treated with control and p63 RNAi oligos. Fixation and staining with the indicated antibodies was performed at 48 hours post-transfections. Note that the laser settings of the C/EBPδ image of p63-inactivated cells had to be lowered considerably due to extemely strong fluorescence. **C**. Chromatin Immunoprecipitation analysis of the C/EBPδ-1 Kb (Indicated by the square box), using HaCaT cells (Left Panel) and the indicated antibodies: NF-YB, p63 (Santa Cruz H137; Dako 4A4) and control -Ctl- anti-Flag antibodies (Sigma). Right Panel, ChIPs with primary KCs, anti-p63 4A4 (Dako), anti-p63α specific polyclonal [19], anti-p53 (Ab7 Calbiochem). As a control, ChIPs from KCs were used to amplify the α-globin promoter. In the bottom part, the p53 and p63 sites at -1000 of the human C/EBPδ promoter are shown, in an area of conservation with the mouse gene. HBA1 is the human α-globin promoter.

We performed RNAi inactivation of p63 in human HaCaT cells followed by gene expression profiling with the Affymetrix platform [19]; one of the genes that was specifically increased under these conditions was C/EBPδ. This gene was similarly found to be affected in primary keratinocytes in Ref. [18]. We confirmed this data by performing RT-PCR to examine the mRNA levels of C/EBPδ, as well as two other members of the family. Upon p63 inactivation, a strong increase was specifically obeserved for C/EBPδ, but not for C/EBPα, while C/EBPβ showed a modest increase (Fig. 1A). β-actin, an invariant mRNA, was used to normalize samples. ΔNp63 was decreased, as expected (Fig. 1A). The pattern was also seen after RNAi inactivation of p63 in primary human keratinocytes (KCs, Fig. 1A, Lower Panel). In this setting, we also checked the protein levels of C/EBPδ by immunofluorescence: Fig. 1B shows that under normal growth conditions, KCs are weakly but uniformly stained with anti-p63 and anti-C/EBPδ antibodies; removal of p63 by RNAi transfections (Central Panels) lead to a substantial increase in the C/EBPδ staining (Left and Merge Panels). To ascertain whether C/EBPδ repression is a primary event, we perfomed ChIP assays, using multiple anti-p63 and control (anti-Flag and anti-NF-Y) antibodies. The anti-p63 antibody we produced and purified recognize all isoforms of p63 [See Ref. [17] for details]. Scanning the human C/EBPδ upstream region, we identified a cluster of potential p53/p63 binding sites around 1 Kb upstream from the transcriptional start site. These sites are in a cluster that is conserved with the mouse sequence (Fig. 1C, Lower Panel). ChIP analysis with HaCaT and primary KC chromatin showed strong positivity with the two p63 antibodies (Fig. 1C). Because HaCaT cells contain two p53 alleles mutated in the DNA-binding domain [21], hence generating proteins presumably incapable of DNA-binding, only in primary cells did we include an antibody against p53: p53 was as positive as p63, an indication that both proteins are bound to the C/EBPδ promoter (Fig. 1C). As a control, we amplified a region of the human α-globin gene, which was devoid of any binding with any of the binding analyzed (Fig. 1C, Upper Panel). We conclude that C/EBPδ is under direct regulation of p63 in human keratinocytes.

### C/EBPδ is regulated upon keratinocytes differentiation

**Figure 2:**
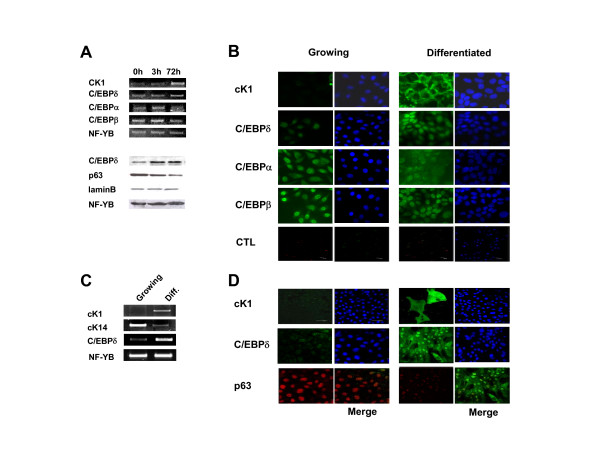
**Regulation of C/EBPδ in HaCaT and primary keratinocytes**. **A**. Upper Panel. RT-PCR analysis of the C/EBPs in HaCaT cells before and after 3 and 72 hours in differentiation medium. cK1 was used as control for differentiation, NF-YB as internal control. Lower Panel. Western blot analysis of HaCat extracts with the indicated antibodies. **B**. Immunofluorescence analysis of growing and differentiated HaCaT [72 hours] using the indicated antibodies. cK1 was used to control for differentiation. Ctl refers to cells stained with the rabbit secondary antibody only. **C**. RT-PCR analysis as in A, except that primary human keratinocytes were used, before or after 3 days in differentiation medium. cK1 and cK14 were used as markers of differentiation. **D**. Same confocal experiments as in B, using human primary keratinocytes.

The role of C/EBPδ in skin differentiation is unknown. As a first step to shed light on this, we wished to know whether it is regulated during differentiation models of human keratinocytes. HaCaT cells can be differentiated upon withdrawal of serum and calcium addition: the cells stop growing, alter their morphology, and express several markers of suprabasal keratinocytes. We checked the levels of C/EBPs by RT-PCR analysis at two times following the differentiation stimulus: early -3 hours- and late, 3 days. The results are shown in Fig. 2A, Upper Panels: the Keratin 1 -cK1- marker was increased, a very modest increase of C/EBPδ, and no change of C/EBPβ or C/EBPα was scored; the invariant histone-like NF-YB was used as an internal control. An increase in C/EBPδ protein level was seen in Western blots (Fig. 2A, Lower Panels) p63 was checked and showed a concomitant reduction. Protein levels were normalized with NF-YB and laminB. Immunofluorescence analysis confirmed that C/EBPδ is increased after differentiation, in contrast to C/EBPα and C/EBPβ (Fig. 2B). cK1 was used to monitor differentiation (Fig. 2B and 2D). Similar experiments with KCs showed a greater increase of C/EBPδ at the mRNA level in RT-PCR (Fig. 2C) and at the protein level in immunofluorescence (Fig. 2D). mRNA levels of cK14 decreased after differentiation, as expected (Fig. 2C). We conclude from these data that C/EBPδ is subject to regulation during differentiation of HaCat cells and primary human keratinocytes, suggesting that it might play a role in the process.

### C/EBPδ is differentially expressed in human skin

**Figure 3:**
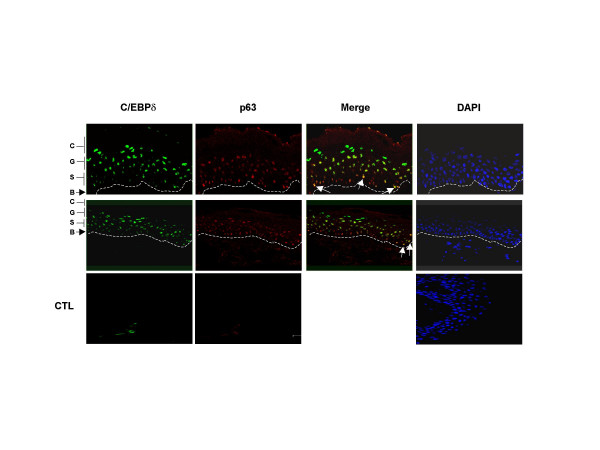
**Regulation of C/EBPδ in human skin**. Fixed samples of two representative human interfollicular skin sections were double stained with anti-p63 4A4 and the C/EBPδ antibodies. Analysis was performed by Immunofluorescence Confocal microscopy. Arrows in the merge Panel indicate nuclear staining of cells that are positive for both antibodies, in the basal layer. Yellow signals in the spinous layer refer to cells co-expressing the two factors. B, basal layer; S, spinous layer; G, granulous layer; C, corneum stratum. In the CTL Panels, we stained a skin section only with mouse and rabbit secondary antibodies used above.

Differential expression in keratinocytes cultured *in vitro *suggests that C/EBPδ might be differentially expressed in the skin. To verify whether this is the case, we evaluated human skin sections of healthy individuals by immunofluorescence confocal microscopy, double staining with p63 and C/EBPδ antibodies. Fig. 3 shows representative immunofluorescence images from two sections. Consistent with previous reports, p63 is strictly confined to nuclei of epidermis and abundant in the basal layer, with expression progressively fading in spinous cells and absent in the granular and corneum strata. C/EBPδ staining is absent in the derma and confined to keratinocytes: it is nuclear and maximal in the granular layer and overlappping with p63 in the spinous layer (Yellow staining in the Merge Panel). Interestingly, C/EBPδ also shows co-staining in some, but not all, cells of the basal compartment of interfollicular skin (Indicated by arrows in the Merge Panel of Fig. 3). Parallel staining with secondary anti-mouse and anti-rabbit antibodies were essentially negative, ensuring that the signals observed above are specific. In conclusion, C/EBPδ is keratinocyte-restricted and clearly regulated in human skin, reinforcing the idea that it is involved in regulating genes during differentiation of keratinocytes. Furthermore, the expression patterns suggest that p63 and C/EBPδ may influence their reciprocal levels by transcriptionally regulating each other.

### Regulation of ΔNp63 by C/EBPδ

**Figure 4:**
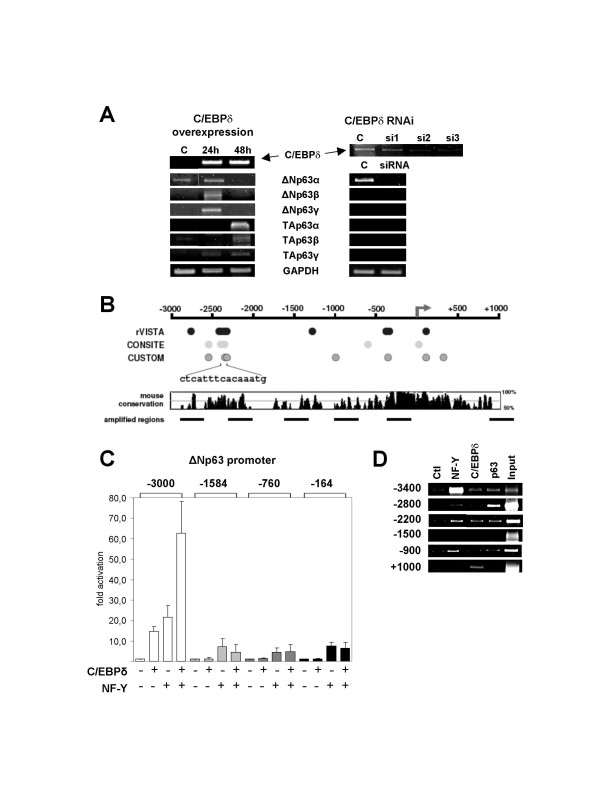
**C/EBPδ is bound to and regulates the ΔNp63 promoter**. **A**. Left Panel. Human KCs were transfected with mouse C/EBPδ. RNAs were extracted at the indicated time and RT-PCR is shown for the indicated genes: controls (mC/EBPδ, GAPDH) and different p63 isoform (upper Panels). Right Panel. RNAi of C/EBPδ with three different oligos. C/EBPδ mRNA was checked for inhibition. **B**. Bioinformatic analsysis of the ΔNp63 promoters for C/EBP sites with different algorithms: a cluster of sites commonly detected at position -2400 is shown. Analysis was performed with rVista [63], CONSITE [64] and CUSTOM. Shown are also mouse conservation and a schematic representation of the amplified region. **C**. Transfection analysis of ΔNp63 constructs [22] without and with NF-Y and/or C/EBPδ. **D**. ChIP analysis of the upstream regions of ΔNp63 with the indicated antibodies, using chromatin from primary KCs.

To study the role of C/EBPδ on p63 expression, we overexpressed C/EBPδ in primary keratinocytes. RT-PCR analysis of p63 isoforms is shown in Fig. 4A (Left Panels). ΔNp63α, the only detectable isoform in mock transfected cells [15-19], increases at 24 hours; surprisingly, ΔNp63β and ΔNp63γ were strongly induced at the same time point. None of the TA isoforms were initially detectable, but they became apparent after 48 hours. In parallel, we inactivated C/EBPδ by siRNA interference in the same cellular setting, using three different oligonucleotides: Fig. 4A (Right Panels) shows that using two of these siRNAs the endogenous C/EBPδ drops to low levels in RT-PCR analysis; oligo 2 was then used for further analysis of the p63 isoforms upon C/EBPδ removal. ΔNp63α indeed decreased dramatically, while none of the other isoforms was expressed, confirming the role of C/EBPδ in ΔN regulation. These data indicate that the ΔN -and TA- p63 promoters are potentially under control of C/EBPδ in keratinocytes.

To test this, we searched for C/EBP sites by using different algorithms in the human ΔNp63 promoter, and found a consensus of two juxtaposed boxes at -2400, in a region that shows considerable homology in the mouse gene (Fig. 4B). This led us to verify the activation of the ΔNp63 promoter using Luciferase constructs of different lengths, from -3000 to -200 relative to the transcriptional Start Site. As the promoter is known to be dependent from NF-Y, a ubiquitous activator [22,23], we included it in our cotransfections assays as a positive control. These experiments were performed in U2OS, which provide a background that is free of the possible dominant self-regulating activity of p63 on its own promoter [22]. We found that NF-Y and C/EBPδ singularly activated the -3000 contruct (Fig. 4C). Remarkably, addition of the two TFs together yielded a clear cooperative effect. However, shorter constructs (-1300, -400 and -200) did not show any significant increase upon C/EBPδ overexpression, while still retaining some NF-Y inducibility, most likely due to core promoter CCAAT boxes detailed in Refs. [22] and [23]. The decrease in NF-Y inducibility with respect of the -3000 construct is probably due to a functional CCAAT box binding NF-Y in this region (See ChIP below). The simplest explanation for these results is that the -2400 C/EBP sites are vital for induction. To confirm that the activation observed is a primary phenomenon, we performed scanning ChIPs analysis on different regions of the C/EBPδ promoter in human KCs; Fig. 4D shows that C/EBPδ and NF-Y are found in the upstream region, in at least three different positions. Note that predicted and actual sites do not always ovelap (Fig. 4C and 4D). Taken together, these data indicate that (i) C/EBPδ binds and activates the ΔNp63 promoter in keratinocytes, (ii) point at the -2400 binding sites as important for regulation, (iii) confirm that NF-Y is a *bona fide *activator of the ΔNp63 promoter.

### Consequences of C/EBPδ overexpression/inactivation on expression of p63 targets

**Figure 5:**
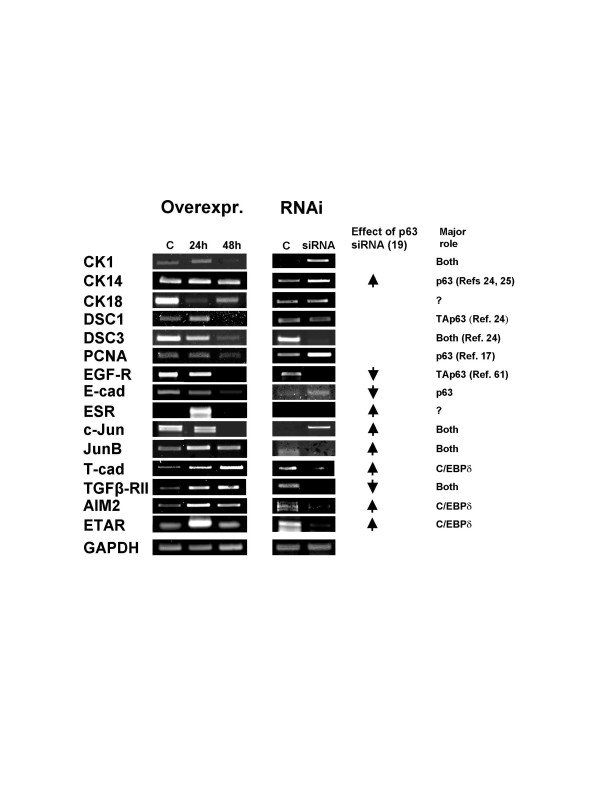
**Overexpression and functional inactivation of C/EBPδ in primary KCs**. Human KCs were transfected with mouse C/EBPδ. RNAs were extracted at the indicated time and RT-PCR is shown for the indicated p63 target genes (Left Panels). In the Right Panels, C/EBPδ inactivation was performed in human KCs with siRNA oligonucleotide 2 of Fig. 4 and with a scramble oligo, in parallel. RT-PCR analysis of the genes in Left Panels is shown. The increase of c-Jun in RNAi-treated cells can only be visualized by low cycles RT-PCR, where a signal from the control oligo RNA is not detectable. On the Right part of the Figure, the effect of RNAi inactivation of p63 is depicted, according to published data and to Ref. 19.

The finding that C/EBPδ regulates p63 prompted us to ascertain whether it would affect the expression of some of p63 targets recently identified, in overexpression and RNAi inactivation experiments in human primary keratinocytes (Fig. 5). We also analyzed by RT-PCR markers of differentiation, such as cK1 and cK14, and Desmocollin (DSC) 1 and 3, important for keratinocyte biology and presumptive C/EBPδ targets [24,25]. C/EBPδ overexpression lead to variations in all genes tested, with the exception of cK14. PCNA, a marker of cell proliferation and itself a p63 target [17] decreased modestly after 48 hours, suggesting a slowdown, but not a stop in proliferation. DSC1, but not DSC3, was activated by C/EBPδ overexpression. Essentially all p63 targets identified in our recent screenings varied in expression, some decreasing, others increasing. Interestingly, some of these genes showed a response at 24 hours, others -EGF-R, c-Jun, E- and T-cadherin- a delayed one.

RT-PCR analysis of keratinocytes in which C/EBPδ was inactivated showed an equally dramatic change in gene expression. In most cases, there is a perfect match with overexpression: genes that are activated, 24 or 48 hours after C/EBPδ transfections are down-regulated by RNAi, consistent with a positive role of C/EBPδ in their regulation. The exception is ESR, whose undetectable levels could not be further decreased by C/EBPδ inactivation. The genes that are repressed in overexpression are increased by RNAi, except EGF-R and Desmocollin 3, in which a dominant role of p63 can be envisaged. In summary, the C/EBPδ RNAi and overexpression experiments are consistent with this TF being important for the expression of many p63 targets.

### p63 targets are bound by C/EBPδ *in vivo*

**Figure 6:**
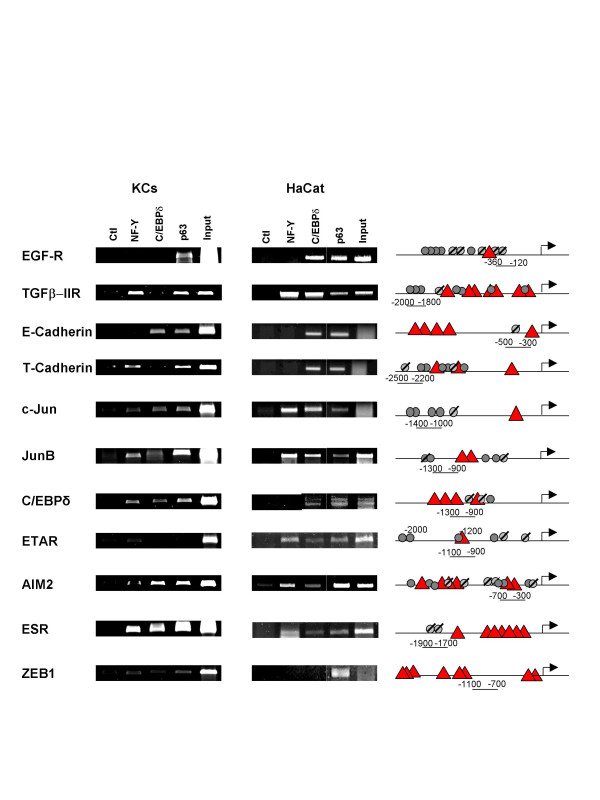
**C/EBPδ binds to p63 targets**. ChIP analysis of NF-Y, C/EBPδ and p63 on different genes targeted by p63 in HaCat cells (Right Panels) and in human primary KCs (Left Panels). The regions considered were described for p63 [19] and are detailed in the cartoon. The analysis of C/EBP (Triangles) consensus sequences according to the three algorithms used in Fig. 4 is depicted. Circles refer to the p63 consensus recently described by Orrt et al [65]. Slashed circles refer to p53 consensus. The position of the amplicons is indicated.

To verify whether the effect of C/EBPδ on expression of the p63 targets analyzed above is a primary event, we performed ChIP analysis with an anti-C/EBPδ antibody in primary KCs and in HaCaT cells. The regions analyzed were the same that showed p63 binding [Ref. [19]. Fig. 6]. Essentially all targets are bound by C/EBPδ, both in HaCaT and in primary KCs, the only exception being Zeb1. Except c-Jun and ETAR, these regions contain one or more C/EBP consensus sequences, as defined by analyzing the common sites derived by the use of the CONSITE, rVISTA and CUSTOM algorithms used in Fig. 4; some are in core promoters, others in upstream locations. The pattern of p63 is somewhat similar, with one target -ETAR- absent in primary KCs. NF-Y sites are found in expected locations, except for E-cadherin, which was shown to be a positive promoter for NF-Y. Note that NF-Y binding on C/EBPδ in primary keratinocytes differs in Fig. 1 (not present) and 6 (present). Individual variations might account for these discrepancies, as we have also noticed differences in positivity in p63 among keratinocytes derived from individual donors with respect of some of the targets [S.B. M.A.V., R.M. unpublished observation]. These exceptions notwithstanding, we conclude that the majority of p63 targets are bound by C/EBPδ *in vivo *in primary and immortalized keratinocytes.

### C/EBPδ-p63 targets are regulated during differentiation

**Figure 7:**
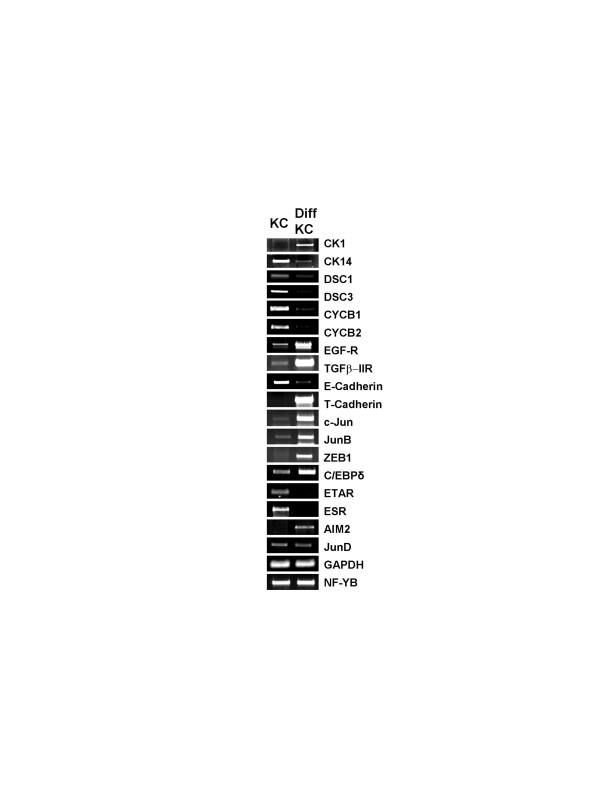
**Regulation of C/EBPδ targets during differentiation**. The targets of C/EBPδ are evaluated in primary KC before and after 72 hours of differentiation by semi-quantitative RT-PCR analysis.

The reciprocal roles of p63 and C/EBPδ for keratinocyte differentiation suggests that coregulated genes would be differentially regulated during this process. We checked their expression levels by RT-PCR analysis in primary keratinocytes induced to differentiate (Fig. 7). The process was monitored as in Fig. 2 with cK1 and cK14 -the former increasing, the latter decreasing- and normalized with GAPDH and NF-YB RNAs. All genes showed remarkable changes. In particular, the coregulated JunB and c-jun varied, while JunD did not. In keeping with the increase in C/EBPδ levels after differentiation, activated genes -TGFβ-RII, T-Cad, JunB- showed a positive variation, irrespective of p63 expression (See Fig. 2). Other genes behaved dissimilarly with respect to C/EBPδ: E-Cadherin and EGF-R, which are repressed, increased considerably upon differentiation. We conclude that all coregulated targets tested change during differentiation, but this complex process, as in the case of p63, cannot be fully recapitulated through variations generated by a single TF.

## Discussion

The concerted modulation of specific gene expression programs is controlled by transcription factors. In specific pathways of response to external stimuli, one, or a few, TFs play a dominant role in this response. Matters are far more complex in multi-cellular systems involving terminal differentiation and the specification of exquisite features. What emerged is the concept of "master" regulators, whose role is to confer cell identity and to drive gene regulation programs accordingly. In the muscle system, for example, some of these masters -MyoD, Myogenin, MEF2- were identified and their gene regulation programs studied: they apparently show a preference for fellow TFs genes. A robust hierarchical cascade of specific programs is adjusted in such multisteps and multi-functions programs [26]. Interestingly, this network is not restricted to TFs exquisitely expressed in muscle, since ubiquitous, or near ubiquitous TFs are an integral part of the network [27]. Similarly, the recent identification of hundreds of p63 targets in various cell types [Reviewed in [28-30]] highlighted links with TFs that are known, or likely, to play an important role in skin biology [28].

The direct cross-talk between p63 and C/EBPδ, originally emerging from p63 RNAi screenings [18,19] is confirmed by ChIP and C/EBPδ RNAi experiments. The decrease in ΔNp63α by inactivation of C/EBPδ strongly argues in favour of an activating role of C/EBPδ on ΔNp63 transcription, confirmed by overexpression; reporter constructs experiments and ChIPs point at important upstream sites at -2.4 Kb. Interestingly, the synergistic activation observed with NF-Y is a further argument in favour of the physiologic role of this particular member of the C/EBP family in ΔN p63 regulation: NF-Y, itself a poor activator, acts primarily in conjunction with a neighbouring TF [Reviewed in [31]]. In the MHC Class II promoters, for example, only the relevant RFX-5, and not other RFXs, is capable to synergize with NF-Y [32].

An intriguing finding is the differential regulation of p63 splicing isoforms in keratinocytes upon C/EBPδ overexpression. Normally, these cells express exclusively ΔNp63α and it is currently unclear whether TA isoforms are induced upon differentiation *in vitro*, since different results were reported [Reviewed in [33] and [34]]. Overexpression of C/EBPδ led to the appearance of ΔNβ and γ, and -later- of the TA isoforms. Thus, C/EBPδ acts not only to support the activity of the ΔN promoter in general, but, directly or indirectly, to impinge on an isoform switch that modifies the p63 portfolio. ΔNp63β and γ induction was also observed upon activation and differentiation of cells of the corneal limbus [35], and a role of TA isofoms in differentiation has been proposed [34]. The switch could have a far reaching effect on expression of p63 targes, particularly in the upper epidermal layers, since the ΔN and TA isoforms have often quite dissimilar transcriptional effects.

How modification of the splicing is accomplished is unclear, at the moment; it has become recently evident, however, that regulation of transcription and RNA processing are highly coordinated events and that specific transcription factors -and cofactors- are known to play a role in pre-mRNA processing [36]. Interestingly, one of the cofactors involved is CARM1 [37], an arginine methyltransferase which modifies other factors promoting exon-skipping [38]. CARM1 is activated by ΔNp63α [19] and it was shown to be involved in other differentiation systems [39]. Thus, it is possible that p63 regulators, such as C/EBPδ, impact on factors loaded on the ΔN promoter and travel along the gene with PolII complexes to adjust splicing events.

Our results indicate that many, perhaps most of the p63 targets are coregulated by C/EBPδ. An intersection between C/EBPs and p53 family members was noticed before. Typical p53-induced pro-apoptotic targets are not activated in the absence of C/EBPδ in KO mammary cells and anti-apoptotic genes are not repressed [13]. The two factors are apparently engaged in a positive feed-back loop, with many genes commonly controlled: Cyclin D1, Bfl1, SGP2, Gas1, Bak and IGFBP5. In addition, expression of C/EBPδ is induced upon Vitamin D treatment in prostate cells, leading to a dramatic arrest in clonal expansion [40], presumably through VDR, another p63 target [41]. Given the pivotal role of p63 in prostate development [42], the interplay between p63 and C/EBPδ could be both direct and indirect, through VDR. Note that the overlap between p53 and p63 targets in the skin is large, but not absolute [17,18].

HaCaT cells are an epithelial line derived from the back of adult human skin that spontaneusly underwent immortalization in vitro [43]; they exhibit normal differentiation and have provided a valuable tool for studying regulation of keratinization in human cells. The interpretation of data concerning p63 in HaCaT is often complicated by the presence of two mutated p53 alleles [44], which are known to impinge on the p63 pathways. This is apparently ot the case for C/EBPδ, but for other p63 targets we are analyzing, there are dramatic differences in regulation between HaCat and KCs (S.B., R.M, in preparation). In light of the overlap between the targets of the two TFs, this implies that a mutated p53 might subvert the tumor suppressive and pro-differentiation role of C/EBPδ.

Based on current knowledge derived mostly from RNAi experiments, a tentative view of the influence of p63 and C/EBPδ is presented in Fig. 5. The picture is complicated by the effect of p63 inactivation through C/EBPδ RNAi and by the differential role of TAp63 [45] activated at late time points. With two exceptions, EGF-R and DSC3, all genes show a consistent behaviour in overexpression and RNAi. Genes that are repressed by p63, ETAR, AIM2, T-Cadherin, JunB, and should be up-regulated by p63 decrease, are rather decreased, an indication that they are mostly rely on C/EBPδ. Some of these -AIM2, T-Cadherin, JunB- limit growth. A role of C/EBPδ as a tumor suppressor was established by the finding that knock-out MEFs show genomic instability, impaired contact inhibition and reduced serum dependance [46]. For the p63-repressed c-Jun, up-regulation by C/EBPδ could either be because of removal of p63 or repression by C/EBPδ, as indicated by overexpression at late time points. As for genes activated by ΔNp63, such as EGF-R, TGFβ-RII and E-cadherin, the latters could be under joint control; EGF-R, which drops after C/EBPδ and p63 RNAi, could decrease after C/EBPδ overexpression at 48 hours through the increase of TAp63 isoforms, shown to be strong repressors of the EGF-R promoter. This would be consistent with a growth control and pro-differentiative role of both TAp63 and C/EBPδ. Similarly, removal of the repressive ΔNp63 [See Ref. [19]] by C/EBPδ RNAi is consistent with an increase in PCNA mRNA and with a small, late decrease in overexpression. In conclusion, it is clear that coregulation of p63/p53 and C/EBPδ is part of a larger program that controls cell-cycle progression and differentiation. To reconstruct the network, it will now be necessary to obtain unbiased information about the panel of C/EBPδ targets in keratinocytes, through the use of RNAi and ChIP on chip technology.

C/EBPδ belongs to a family of TFs important for several differentiation pathways, including adipocytes, liver and haematopoietic lineages [7]. Specifically, it has been associated to the early phases of adipocyte differentiation, together with C/EBPβ. It is induced upon several environmental changes that lead to growth arrest [47-51] and serum withdrawal in mammary cells and lung epithelia cells [52]. Overexpression leads to growth arrest in mammary and prostate epithelial cell lines [53-55]. The growth arrest features are common to other C/EBPs [Reviewed in [56]]. Our findings that C/EBPδ is up-regulated during cell-cycle exit and differentiation of immortalized HaCaT and primary keratinocytes, and the decrease in expression of PCNA, a proliferative marker, are in agreement with these results.

Nothing is known about C/EBPδ expression during epidermal development of mouse skin. Despite careful examination, the available antibodies are unable to stain mouse cells in immunofluorescence analysis [B.T, R.M., unpublished]. In humans, little variation of expression of C/EBPδ was detected in the different phases of maturation of skin annexes, the hair follicle and the sebaceous gland, unlike other members of the family [57]. At the opposite, we observe here that C/EBPδ staining is not uniform in human interfollicular skin, suggesting that the reciprocal interplay with p63 is complex. Co-expression is seen in selected basal cells, which is in line with the activating role of C/EBPδ on the ΔN promoter; in other cells of the same layer, strongly positive for p63, C/EBPδ fades: this is also consistent with the negative role of p63 on C/EBPδ expression. There is a coexpression and a balance in the spinous layer, and in the upper granular layer, with cells exiting from a proliferation status and terminally differentiating, C/EBPδ is prominent. In transfection assays, Desmocollin 3, which is expressed in basal cells, is transactivated by C/EBPδ and C/EBPβ, the suprabasal Desmocollin 1 by C/EBPδ and C/EBPα [24]. We confirm that both these genes require C/EBPδ expression, as determined by RNAi experiments. Thus our results are consistent with C/EBPδ playing a dual role in skin differentiation, both in very early and late stages. In the former, it could limit, through its growth suppressive properties the growth potential of early progenitors. In the later stages, it may coordinate cell-cycle exit and induce differentiation markers. Our observations could now be extended in other systems, notably in the mammary gland and in prostate, in which both TFs have been singularly been shown to play key roles.

One caveat to the C/EBPδ role in skin is the fact that KO mice have no apparent alterations. However, this might be due to redundancy with C/EBPβ: it was reported that C/EBPβ is expressed in the cytoplasm of basal keratinocytes and becomes nuclear in the spinous layer [58]. C/EBPβ KO mice have skin hyperplasia with downplay of keratin 1 and 10 expression [59] and C/EBPβ is important for keratinocyte survival [60]. Of particular significance are the data recently reported by the group of Vinson on a transgenic model expressing a dominant negative C/EBP -termed A-C/EBP- in basal keratinocytes: these mice have hyperplasia of the basal epidermis and increased apoptosis of the upper layers [61]. p53 and pro-apoptotic markers are induced and C/EBPβ dramatically reduced; as A-C/EBP is not C/EBPβ-specific, C/EBPδ might contribute to the observed phenotype. Given the well known complexity within this family, through homo- and heterodimerizations, production of dominant negatives through the use of internal AUG within C/EBPβ, much work lies ahead to establish their role in the different layers of epidermis.

## Conclusion

We identified a direct and mutual link between p63 and a member of the C/EBP family of transcription factors, C/EBPδ. The following relevant findings are the reported: (i) C/EBPδ expression is confined to keratinocytes and it is up-regulated in cells committed to differentiation; it is increased in *in vitro *differentiation systems, both of HaCaT and primary keratinocytes. (ii) C/EBPδ activates transcription of the ΔNp63 promoter, and it appears to be part of the mechanisms that control p63 splicing. (iii) It is involved in regulation of other p63 targets regulated during differentiation, as verified by RNAi, overexpression and ChIP assays. We therefore suggest that the mutual link between these TFs is important for the correct differentiation of keratinocytes.

## Methods

### Cells and culture conditions

First passage primary human keratinocytes -KCs- were derived from breast of healthy individuals and grown on a feeder-layer of lethally irradiated 3T3 cells in DMEM F12 added of Insulin (5 μg/ml), EGF-R (10 ng/ml) hydrocortisone (0.4 μg/ml), T3 (2 nM), Cholera toxin (0.1 nM) and transferrin (5 μg/ml). HaCaT were grown in DMEM or in low calcium medium when assayed for differentiation, which was added with 1.4 mM CaCl_2 _in 0.1% serum conditions. Primary KCs were differentiated by adding CaCl_2 _(1.4 mM final concentration) in the presence of 10% foetal calf serum.

### RT-PCR and transfections

HaCaT cells were transiently trasfected using Oligofectamine or Lipofectamine 2000 (Gibco-BRL, USA) for 3 hours with 150 ng/cm^2 ^of human p63 siRNA oligonucleotide (ACAATTTCATGTGTAACAGCA) which targets aminoacids 265–272 in the central DNA-binding domain of p63. After incubation overnight in DMEM, transfection was repeated for 3 additional hours. RNA was extracted from HaCaT cells using an RNA-Easy kit (Qiagen). 2.5 × 10^5 ^first passage primary human keratinocytes from healthy individuals were similarly transfected with Nucleofector (Amaxa, D) according to the Manufacturer' conditions with the off-target siRNA control oligos (5'-AUGAACGUGAAUUGCUCAA-3', 5'-UAAGGCUAUGAAGAGAUAC-3', 5'-AUGUAUUGGCCUGUAUUAG-3', 5'-UAGCGACUAAACACAUCAA-3'; Dharmacon D-00181001), or with three different oligonucleotides targeting human C/EBPδ (S1: GAUGCAGCAGAAGUUGGUGuc; S2: GACUCAGCAACGACCCATuu; S3: GGAAAAGACUGAGCAUGCUuu).

RNA was extracted 24 or 48 hours after transfections. For cDNA syntesis, 4 μg of RNA were used with M-MLV-RT kit (Invitrogen, USA). Semi-quantitative PCR analysis were performed with specific primers [see additional file 1].

In Figure 4, 2 × 10^5 ^U2OS were transfected with Lipofectamine (Gibco-BRL) using 1,2 μg of reporter plasmids, 200 ng of C/EBPδ, 70 ng of the NF-YA, NF-YB, NF-YC plasmids, and carrier for a total DNA concentration of 2 μg. Six independent transfections in duplicate were performed.

### Chromatin immunoprecipitations

ChIP analysis were carried out with the method described in Ref. [19] with an anti-C/EBPδ antibody (Active Motif #39006), anti NF-YB (Diagenode), anti-p53 (Ab7, Calbiochem) and anti-p63 abs (SantaCruz SC137, 4A4 DAKO, Diagenode). For the oligos used see additional file 1.

### Western blot and Immunofluorescence

Western blot analysis was performed using standard procedure with a Pierce secondary antibody and detection system. Skin sections derived from thighs of healthy donors are shown in Figure 3. Cryopreserved human skin sections were fixed in 4% fresh paraformaldehyde for 10' and incubated o/n with the primary antibodies anti-p63 [17] and anti-C/EBPδ (Active Motif #39006). After 1 hour incubation with the fluorocrome-conjugated secondary antibodies, the slides were stained with DAPI and mounted in Vectashield. Fluorescence was analysed with a Leica confocal microscope.

### Note added in proofs

After submission of this manuscript, Barbaro et al. (J. Cell Biology 177, 1027–1049, 2007) reported that C/EBPδ and p63 are coexpressed in stem cells of the corneal limb epithelium and share common gene targets. These authors also resported that C/EBPδ binds to the ΔNp63 promoter.

## Authors' contributions

SB performed the C/EBPdelta and p63 RNAi and overexpressions, RT-PCR, ChIPs. BT performed the original p63 screenings in HaCaT, validated C/EBPδ as a target and performed confocal analysis of Figures 2 and 3. MC performed the bioinformatic and transfection analysis of Fig. 4. DA and CC purified primary human keratinocytes. RAR and SS prepared the ΔNp63 promoter constructs. MAV analyzed the Affymetrix profiling data. RM wrote the manuscript.

## Competing interests

The author(s) declares that there are no competing interests.

## Supplementary Material

Additional file 1 Primers for RT-PCR and ChIP analysis. The oligos used for semi-quantitative RT-PCR and ChIP analysis. Click here for file
