# Supplementary material for: Reciprocal regulation of p63 by C/EBP delta in human keratinocytes
Source: BMC Mol Biol. 2007 Sep 28;8:85. doi: 10.1186/1471-2199-8-85 (PMC2148061; doi:10.1186/1471-2199-8-85)
Supplement: Additional file 1 — Primers for RT-PCR and ChIP analysis. The oligos used for semi-quantitative RT-PCR and ChIP analysis. [file 1471-2199-8-85-S1.doc]

**Supplementary 1**

**Primers for RT-PCR analysis.**

TAp63for: 5’-TTAGCATGGACTGTATCCGC-3’;

DNp63for: 5’- CCAGACTCAATTTAGTGAGC-3’;

TADN α rev: 5’-ACTTGCCAGATCATCCATGG-3’;

TADN γ rev: 5’-AAGCTCATTCCTGAAGCAGG-3’;

TADN β rev: 5’-TCAGACTTGCCAGATCCTG-3’;

C/EBPα: 5’-ttggtcaaggccatgggca-3’, 5’-tgcagctctggaggaagc-3’;

C/EBPβ: 5’-gcgcgagcgcaacaacatc-3’, 5’-tgcttgaacaagttccgcag-3’:

C/EBPδ: 5’-cctcccaaaatgctgggattac-3’, 5’-ttccaggtctacggaagcagtg-3’;

NF-YB: 5’-AGGTGCCATCAAGAGAAACG-3’, 5’-TGTTGTTGACCGTCTGTGGT-3’;

β-actin : 5’-TGGGTCAGAAGGATTCCTATGT-3’, 5’-CAGCCTGGATAGCAACGTACA-3’;

cK1: 5’-GGACATGGTGGAGGATTACCG-3’, 5’-TGCTCTTCTGGGCTATATCCTCG-3’; cK14: 5’-TGGCCGCGGATGACTTC-3’, 5’-CTCGCTCTTGCCGCTCTG-3’;

E-Cadherin: 5’-gggagcgttaggaaggaatc-3’, 5’-AGGATGGTGTAAGCGATGGC-3’;

T- cadherin: 5’-TTCAGCAGAAAGTGTTCCATAT-3’, 5’-GTGCATGGACGAACAGAGT-3’;

ZEB1: 5’- TGCCAATAAGCAAACGATTC-3’, 5’-TGAGGTCTTTTACCTGTGTG-3’

EGFR : 5’-GAGAGGAGAACTGCCAGAA-3’, 5’-GTAGCATTTATGGAGAGTG-3’;

TGFβ-RII:5’-TGGAGAAAGAATGACGAGAAC-3’,5’AAGATGATGTTGTCATTGCACTC-3’;

CYCB1 : 5’-CACTTCCTTCGGAGAGCATC-3’,5’-CAGGTGCTGCATAACTGGAA-3’; CYCB2 : 5’-CAGTTCCCAAATCCGAGAAA-3’ , 5’-TCTGAGACAAGCAGGAAGCA-3’; AIM2 : 5’-CAGATGGTGGCCCAGCAGG-3’,5’-gaactccagatgtcagctg-3’;

C-Jun:5’-GCATGAGGAACCGCATCGCTGCCTCCAAGT-3’ ,5’-GCGACCAAGTCCTTCC CACTCGTGCACACT-3;

JunB : 5’-ccagtccttccacctcgacgtttacaa-3’,5’-gatgccatcccgcccacttt c -3’;

JunD : 5’-cgtgccgagcttcggcgacagc-3’, 5’-gctggcagccgctgttgacgtgg-3’;

ER : 5’-cgggactgcggtaccaaatat-3 , 5’-ctggcgcttgtgtttcaacat-3’;

PCNA : 5’-cctgtgcaaaagacggagtgaa-3 ,5’-caccgttgaagagagtggagtg g-3’ ;

DSC1 : 5’- GTGCCAGCGTCTTCTTAGCCGC-3’ , 5’-GCCTGAAGATGAGAAGGAACTC G-3’;

DSC3 : 5’- AGGTCTCGCTCTCGGCACCCTC-3’ , 5’- GGCTTCACCAGCACGACTGAAG-3’.

**Primers for ChIP analysis.**

C/EBPδ promoter: 5’-cctcccaaaatgctgggattac-3’; 5’-tccaggtctacgga agcagtg-3’.

ΔNp63 promoter: -3.4 Kb: 5’-aatggagccattgacatcggta-3’ , 5’-tgtttcagac acagcgtgaaagg-3’ ,-2.8 Kb: 5’-ctgaggacttgggacattgacg -3’ , 5’-ttga actcagcacaaacatttcca-3’, -2.2 Kb: 5’-gcacggagctcctgtct ccta-3’ , -1.5 Kb: 5’-tgccttctaggcagtgctcatt-3’, 5’ccggatttgcctcccct tta ctt 3’; -800: 5’-ggccagattctacatgaatgttggt-3’, 5’-accaccggtcccg ttaac ttac-3’; -100: 5’-tctgtaaatcgtggtggtggtg-3’; 5’-aggaggcgggac tcttc tcttt-3’; +100: 5’-ggggagtgttgtggagagaga-3’, 5’-agcaagcctgct tgaa tgtttt-3’.

junB promoter 5’- cagtcgtggaagatccagcagt-3’ , 5’- gctgcatgacccatta aattgc-3’;

C-jun promoter: 5’- aaataaccgcggaaaaggaacc-3’ , 5’- cagatgggaacaagcgtgta gg-3’;

E-cadherin promoter: 5’- catggctcacacctgaaatcc-3’ , 5’- agtacaggtgcacaccacca-3’ ;

T- cadherin promoter: 5’- gggagcgttaggaaggaatc-3’ , 5’- gagttctcggctgcattttgt -3’ ;

ETAR promoter: 5’- tgcctgcactaagaggatgg-3’ , 5’- CGCAAGAGcgtcctaac ctt-3’ ;

ESR promoter: 5’- cgagcacattccttccttcc-3’ , 5’- gcaatcctcatctccctgct-3’;

EGFR promoter: 5’- ctcctcctcctctgctcctc-3’ , 5’- gacgaggtggcctgtcgtc-3’;

AIM2 promoter: 5’- cttggctgactgctgaccctta-3’ , 5’- tgaaaatgagacccaag cgaaa-3’;

TGFβ-RII promoter: 5’- TGGAGAAAGAATGACGAGAACA-3’ , 5’- AAGATGATGTTGTC ATTGC ACTC-3’.

HBA1 promoter : 5’- CGGTTCCAGCTATTGCTTTGTT-3’ , 5’- CGGGATCGGGGAACACAC - 3’
